# Thickness Profile of Donated Corneas Preserved in Optisol-GS versus Sinasol: An Ex-vivo Study

**DOI:** 10.18502/jovr.v18i4.14546

**Published:** 2023-11-30

**Authors:** Fatemeh Javadi, Bahareh Kheiri, Amir Rezaeian Akbarzadeh, Mozhgan Rezaei Kanavi

**Affiliations:** ^1^Ophthalmic Research Center, Research Institute for Ophthalmology and Vision Science, Shahid Beheshti University of Medical Sciences, Tehran, Iran; ^2^TiamPharma Labs Company, Tehran, Iran; ^3^Ocular Tissue Engineering Research Center, Research Institute for Ophthalmology and Vision Science, Shahid Beheshti University of Medical Sciences, Tehran, Iran; ^5^Fatemeh Javadi: http://orcid.org/0000-0003-4661-4529; ^6^Mozhgan Rezaei Kanavi: http://orcid.org/0000-0002-1497-2260

**Keywords:** Optisol-GS, Sinasol, Corneal Thickness, Visante Optical Coherence Tomography

## Abstract

**Purpose:**

This study aimed to compare the thickness profile and the endothelial cell density (ECD) of donated corneas maintained in Optisol-GS with those preserved in Sinasol over seven days.

**Methods:**

Twenty paired donor corneas were received from the Central Eye Bank of Iran. After recording the osmolarity of each medium, one of each of the cornea pairs was preserved in either Optisol-GS or Sinasol media. Then, slit-lamp biomicroscopy and specular microscopic examinations were performed at the baseline and on day seven. Visante optical coherence tomography (V-OCT) was also performed at 1 hour (h), 24h, 72h, and one week post-preservation. The specular microscopic and V-OCT values were then compared between the two groups.

**Results:**

The mean osmolarity of the Sinasol group was significantly less than the Optisol-GS group (296 vs. 366 mOsm/L, P = 0.0008). The mean central corneal thickness at the measurement points was comparable between the two groups. However, the increase of thickness one week post-preservation in the Sinasol group was remarkably lower than those in the Optisol-GS group ( P = 0.027). The mean ECD was comparable between the groups at the baseline and on day seven. However, the mean change of ECD from baseline to day seven was considerably higher in the Optisol-GS group than in the Sinasol group ( P = 0.019).

**Conclusion:**

Corneal storage in Sinasol over seven days provides better and superior maintenance and preservation of corneal tissue deturgescence and a lower rate of ECD loss over Optisol-GS.

##  Introduction 

The quality of the corneal storage medium is crucial in determining more precise and accurate results when performing a keratoplasty.^[1]^ The main goal in developing corneal storage media is maintaining the functional status of the corneal endothelial layer while inducing corneal deturgescence. Corneal deturgescence is a condition that ensures that the cornea remains relatively dehydrated following the process of corneal preservation, which can contribute to the successful outcome of the tissue transplantation procedure in addition to minimizing the risk of graft failure.^[2,3]^

Among the intermediate types of cold storage media such as Optisol-GS, Dexol, Eusol-C, Cornisol, K-sol, Procell, Cornea ColdⓇ, Chen and Life4C,^[1,4,5,6,7,8,9,10]^ Optisol-GS has been widely used for the storage of donated corneas in the Central Eye Bank of Iran.^[7]^ Recently, a new corneal storage medium, Sinasol (currently named as ZiSol; a joint product of “Research Institute for Ophthalmology and Vision Science, Shahid Beheshti University of Medical Sciences, Tehran, Iran”, “Central Eye Bank of Iran", and “TiamPharma Company, Tehran, Iran"), has been introduced into the market to enhance the accessibility and affordability of corneal storage medium for developing countries. The Sinasol medium has been widely used as an intermediate-term corneal storage medium in Iran since August 2018.^[11]^ Its main composition is almost the same as Optisol-GS except for the omission of some miscellaneous components such as amino acids, adenosine triphosphate precursors, human recombinant insulin, L-glutamine, and vitamins in the Sinasol medium.^[11]^

Through our documentation in our previous study,^[11]^ we demonstrated that Sinasol was as efficient as Optisol-GS in preserving the donated corneal tissues. However, the information on the thickness profiles of the stored cornea was not addressed. Hence, this study was designed to measure the thickness profiles of corneas stored in Optisol-GS versus those held in Sinasol. These profiles were measured using Visante OCT (V-OCT) in order to determine the efficiency of one medium over another in terms of the induction of corneal deturgescence over seven days.

**Figure 1:**
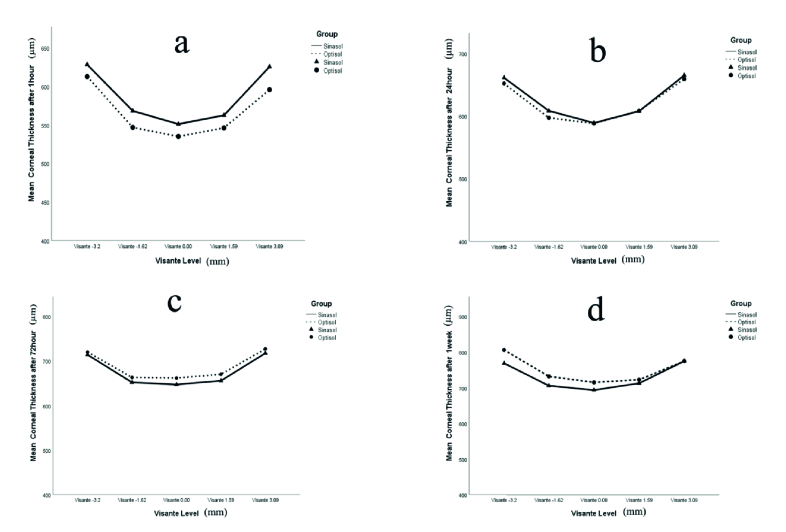
Mean corneal thickness in five settings on Visante OCT for Optisol-GS and Sinasol media at 1h (a), 24h (b), 72h (c) and 1-week (d) post preservation time. The illustrated graphs show that whole corneal thickness in the Sinasol group is higher than the Optisol-GS at the baseline (a), but with an increase in the storage time, the thickness values are higher in the Optisol-GS than the Sinasol group (b-d).

**Table 1:** Mean central corneal thickness in the Sinasol and Optisol-GS groups.


orangeMean central corneal thickness (µm)			
orange**Preservation time**	orange**Sinasol**	orange**Optisol-GS**	orange ***P*** value*
Baseline (1h)	551 ± 53	535 ± 53	0.34
24h	589 ± 32	588 ± 41	0.949
Change from baseline to 24h	38 ± 50.01	53.35 ± 40.28	0.292
***P*** value**	0.003	< 0.001	
72hour	648 ± 32	662 ± 47	0.261
Change from baseline to 72h	96.55 ± 53.99	127.25 ± 52.61	0.076
***P*** value**	< 0.001	< 0.001	
1 week	694 ± 49	715 ± 59	0.274
Change from baseline to 1 week	152.94 ± 41.32	187.31 ± 42.48	0.027
***P*** value**	< 0.001	< 0.001	
	
	
white<bcol>4</ecol>*Based on generalized estimation equation; ** Based on paired t-test

**Table 2:** Mean change of corneal thickness from the central (visante 0.00 mm) towards the peripheral locations (visante -3.20 mm and +3.09 mm) in the Sinasol and Optisol-GS groups.


orange**Mean change of corneal thickness (µm)**				
orange**Visante (mm)**	orange**preservation time**	orange**Sinasol ( n =20)**	orange**Optisol-GS ( n =20)**	orange ***P*** value*
0.00 to -3.20	1 hr to 24hr	4.65 ± 52.13	13.75 ± 49.64	0.575
0.00 to -3.20	1 hr to 72hr	10.65 ± 60.38	19.8 ± 74.16	0.671
0.00 to -3.20	1 hr to 1 week	3.88 ± 50.32	6 ± 49.06	0.578
0.00 to +3.09	1 hr to 24hr	1.75 ± 58.59	10.5 ± 41.99	0.59
0.00 to +3.09	1 hr to 72hr	4.25 ± 48.05	4.6 ± 41.61	0.537
0.00 to +3.09	1 hr to 1 week	6.94 ± 45.67	7.63 ± 44.61	0.369
	
	
white<bcol>5</ecol>*Based on generalized estimation equation

**Table 3:** Corneal endothelial cell densities in the Sinasol against Optisol-GS groups at the baseline and on day 7.


	orange**Groups**		
	orange**Sinasol**	orange**Optisol-GS**	orange ***P*** value*
Mean ECD at baseline (cells/mm^2^)	2918 ± 472	2846 ± 369	0.644
Mean ECD at day 7 (cells/mm^2^)	2615 ± 582	2362 ± 581	0.057
Mean change of ECD from baseline to day 7 (cells/mm^2^)	302 ± 476	488 ± 507	0.019
***P*** value**	< 0.001	< 0.001	
	
	
white<bcol>4</ecol>ECD: Endothelial cell density; * P -value based on Mann-Whitney U; ** P -value based on Wilcoxon

**Table 4:** Components of Optisol-GS vs. Sinasol.


orange**Component**	orange**Optisol-GS ${}^{\text{1,10}}$ **	orange**Sinasol ${}^{\text{11}}$ **
Basic component	TC-199 and MEM-Earle	MEM
Deturgescent agent	Dextran T40	Dextran T70
Buffer	Sodium bicarbonate HEPES	Sodium bicarbonate HEPES
Antibiotic	Gentamicin Streptomycin	Gentamicin Penicillin-Streptomycin
Others	Chondroitin sulfate *L*-glutamine Pyruvate Nonessential Amino Acids 2-mercaptoethanol Ascorbic Acid ATP Precursors Purines Vitamins	Chondroitin sulfate
	
	

##  Methods

Ethical approval for the current study was obtained from the Institutional Review Board of the Central Eye Bank of Iran and the Ethics Committee of the Research Institute for Ophthalmology and Vision Science, Shahid Beheshti University of Medical Sciences, Tehran, Iran (IR.SBMU.ORC.REC.1395.14).

### Osmometry

Before starting the experiment, the osmolarity of all implemented preservative media was measured using a VAPROⓇ Vapor Pressure Osmometer (Wescore Inc., Utah, USA).

### Donor corneas

In this prospective, single-blind ex-vivo comparative study, 40 corneas from 20 seropositive donors were obtained from the Central Eye Bank of Iran. The inclusion criteria were donors with no history of previous eye surgery, a death to storage time of less than 30 h, and a good to excellent cornea rating on the slit lamp biomicroscopy.^[7,11]^ One cornea from each donor was randomly maintained in Optisol-GS, and the fellow cornea was stored in Sinasol at 4 ${}^{\circ }$ C for one week. In order to conceal the brand of the medium being used from the examiner, the media labels were covered, and labels of medium#1 and medium#2 were considered for Optisol-GS and Sinasol, respectively. Specular microscopy and V-OCT were performed on the preserved corneas.

### Slit-lamp biomicroscopic and specular microscopic examinations

Slit-lamp biomicroscopic examinations (Haag Streit, BQ 900, Koeniz, Switzerland) were performed for all samples to exclude cases with previous eye surgeries or endothelial disorders. Whole globes that had very good to excellent endothelial cell ratings were used in the study.

Before performing specular microscopic examinations with the KeratoAnalyzer (EKA-10; Konan Medical Inc., Hyogo, Japan), corneal tissues were warmed for 30 min at room temperature. Images from the central and pericentral areas of the endothelium were captured. The center mode was used to quantify the endothelial cell density (ECD), and all the visible cells per image were counted. Four captured images per cornea were analyzed in each specular microscopic examination, and the mean of the measured ECDs was considered for statistical analyses. The measurements were carried out by a skilled eye bank technician (T. Ch) at the baseline and repeated on day seven. The results were then reassessed by an ophthalmologist/eye bank specialist (MRK).

### V-OCT examinations

The thickness profile of the preserved corneas was obtained using the V-OCT (Carl Zeiss Meditec, Inc., Dublin, CA, USA) at 1h, 24h, 72h, and seven days post-preservation. All the examinations were performed by an expert eye bank technician (T. Ch), and the corresponding results were approved by an ophthalmologist/eye bank specialist (MRK). The device was pre-calibrated, and all measurements were performed at room temperature (20 ${}^{\circ }$ C to 22 ${}^{\circ }$ C).

As previously described,^[11,12]^ the vial containing the cornea was fixed onto the V-OCT system using a custom-designed mount. Tissue thickness measurements were then performed at the vertex center (visante 0.00 mm), pericentral (visante +1.59 mm and -1.62 mm), and peripheral (visante +3.09 mm and -3.20 mm) locations between the Bowman's layer and the endothelial layer while the corneal tissue was immersed in the medium.

For measuring tissue thickness in both media, V-OCT values were adjusted for the refractive index at the pericentral and mid-peripheral locations.^[12,13]^ As the OCT beam was perpendicular to the corneal tissue, the central corneal thickness (CCT) values were not adjusted.^[14]^

### Statistical analyses 

Frequency (percent), mean ± standard deviation, median, and range were used to describe the data. A paired T-test was used to investigate the differences between the groups at the corresponding time periods in terms of their corneal thickness profiles and ECDfor data of normal distribution. When the data distribution was not normal, Mann-Whitney U and Wilcoxon were used as non-parametric tests. A generalized estimating equation was also used to assess the correlation between the two eyes of each donor. SPSS software was used for statistical analyses (IBM Crop. Version 25.0. Armonk, NY). A P -value lower than 0.05 was considered significant.

##  Results

A total of 40 corneas from 20 donors with a mean age of 36.9 ± 13.6 years (ranging between 22 and 62 years) were included in the study. Of these, 14 (70%) donors were male. All corneas were donated as phakic whole globes.

The mean osmolarity of the implemented Sinasol media (296 ± 6 mOsm/L) was significantly lower than that of Optisol-GS (366 ± 7 mOsm/L) ( P =0.0008). Regarding the V-OCT examinations, the mean central corneal thickness (CCT) at all measurement points was comparable between the groups. As shown in Figure 1, the whole corneal thickness in the Sinasol group was higher than the Optisol-GS at the baseline. Still, with increases in the storage time, the thickness values were higher in the Optisol-GS than in the Sinasol group. Additionally, the increase of thickness from the baseline to 1-week post preservation in the Sinasol group was significantly lower than that in the Optisol-GS group ( P = 0.027) (Table 1). As shown in Table 2, there was a significant increase in thickness from the central to the peripheral regions over the one week, but the rate of increase was not remarkably different between the two groups.

Mean ECD significantly reduced over the 7-day storage period in both groups (Table 3). ECD reduction was not remarkably different between the two groups at the baseline and on day seven. However, the mean change of ECD from the baseline to day seven was notably higher in the Optisol-GS than in the Sinasol group ( P = 0.019). Accordingly, based on the specular microscopic results, the rates of decline of ECD in the Sinasol and Optisol-GS groups were 10.4% and 17.2%, respectively.

##  Discussion

The results of the current study revealed that at 1-week post-preservation time, mean corneal thickness was significantly increased in both the central and peripheral parts of the donated corneas stored in both Sinasol and Optisol-GS. This finding may be due to the decrease in the endothelial ionic pump function^[14,15]^ as well as the increase of the endothelial permeability over the maintenance period.^[8]^ However, the corneal swelling in the Sinasol group was lower than those preserved in the Optisol-GS group on day 7, which may be due to the better deturgescence effect of Sinasol over Optisol-GS. Deturgescence activity, or the corneal tissue's capability to remain relatively dehydrated, is performed mainly by the endothelial cells and is required to maintain corneal transparency.^[2,3]^ Therefore, the main goal of developing new corneal storage media has been to encourage the preservation of corneal endothelial cells and the corresponding deturgescence activity during the storage time.^[2,16]^

The main components of the Sinasol solution are almost similar to that of the Optisol-GS solution (Table 4) except for the absence of a few accessory materials, such as vitamins and ATP precursors in the Sinasol, which makes the final cost of Sinasol lower than Optisol-GS.^[11]^ As illustrated in Table 4, dextran and chondroitin sulphate, which are known main components of Sinasol and Optisol-GS, are responsible for eliminating the excess water from the cornea and regulating the stromal hydration over the preservation time through induction of an osmotic effect.^[2,11,15,16]^ Sinasol contains dextran T70 in contrast to Optisol-GS, which contains dextran T40.^[11]^ As dextran T70 is retained in the intravascular space,^[11,17]^ its presence in the Sinasol solution may assist in reducing tissue edema and consequently lowering corneal thickness as opposed to the Optisol-GS that contains dextran T40. It seems that Sinasol preserves the barrier function of the endothelial cells more effectively than the Optisol-GS solution. This finding is in agreement with the results of the study by Parekh et al. ^[10]^ which showed a better preservation of endothelial cells in the Cornea ColdⓇ medium containing dextran T500^[10]^ than the Optisol-GS at the first seven days of storage, and in contrast to the results by Ho et al.,^[18]^ in which the mean CCT on day 7 was remarkably higher in the Cornea ColdⓇ than the Optisol-GS group.

We discovered that the whole corneal thickness at the baseline in the Sinasol group was higher than that of the Optisol-GS group. However, with increasing storage time of up to seven days, the thickness values were higher in the Optisol-GS than in the Sinasol group. One explanation for this observation could be the dissimilar tonicity of the media. The mean osmolarity of the Sinasol solution in our study was 296 ± 6 mOsm/L, which was significantly lower than that of the Optisol-GS (366 ± 7 mOsm/L). It might be a critical factor for initiating rapid corneal deturgescence in the Optisol-GS solution. A similar observation was reported by Nelson et al.^[8]^, in which 10 to 30 min after corneal placement in the Chen (about 305 mOsm/L) and Optisol-GS media, the corneal thickness at the baseline in the Chen group was higher than those maintained in the Optisol-GS.

In our study, mean ECD significantly reduced over seven days of storage in both groups. In addition, ECD reduction was comparable between the two groups at the baseline and at day seven. These findings align with our previous study, which showed that mean ECD at seven days of corneal storage in the Sinasol solution was comparable with those in Optisol-GS.^[11]^ We also found that the mean change of ECD from baseline to day seven was notably higher in the Optisol-GS group than in the Sinasol group. Based on the statistics, it seems that Sinasol provides a lower rate of ECD loss when the storage time of donated corneas is increased.

Sinasol, unlike Optisol-GS and Cornisol, lacks miscellaneous components such as amino acids, adenosine triphosphate precursors, human recombinant insulin, L-glutamine, or vitamins.^[11]^ These supplements were initially advertised as antioxidants or physiological ECs growth modifiers with the aim of preserving corneal endothelial cells and reducing the rate of vacuolization.^[19]^ However, the study of Greenbaum et al.^[20]^ showed no superiority between Optisol-GS (containing supplementary antioxidants) and Dexsol (without the supplementary antioxidants). Moreover, in our prior study,^[11]^ we observed similar performances between Sinasol and Optisol GS on corneal endothelial indices such as EC vacuolation and mortality over two weeks of corneal preservation. Considering the results of the current study, the presence of the antioxidant components in the Optisol-GS could not even make it superior to the Sinasol in terms of preservation of corneal tissue deturgescence or a lower rate of ECD loss.

In the current study, we did not use vital dyes to evaluate the percentage of dead corneal endothelial cells existing within the donated corneas, although based on the specular microscopic results, the rate of decline of ECD in the Sinasol group was less than that in the Optisol-GS. In our previous study,^[11]^ we showed no remarkable difference between the Optisol-GS and Sinasol groups in the percentage of trypan blue-stained dead endothelial cells. However, we are quite certain that the functionality of the remaining endothelial cells can be validated when sustained corneal deturgescence and transparency are achieved.

In summary, our results suggest that over seven days of storage, Sinasol as self-sufficient, cost-effective, and accessible national production can be advantageous over Optisol-GS in terms of induction of a lower whole corneal thickness and a higher corneal deturgescence along with a lower rate of ECD reduction, which is critical for corneal transparency and consequent successful corneal transplantation.

##  Financial Support and Sponsorship

None

##  Conflicts of Interest

None
